# Impact of Environmentally Relevant Concentrations of Bisphenol A (BPA) on the Gene Expression Profile in an In Vitro Model of the Normal Human Ovary

**DOI:** 10.3390/ijms23105334

**Published:** 2022-05-10

**Authors:** Aeman Zahra, Rachel Kerslake, Ioannis Kyrou, Harpal S. Randeva, Cristina Sisu, Emmanouil Karteris

**Affiliations:** 1 Department of Life Sciences, Division of Biosciences, College of Health, Medicine and Life Sciences, Brunel University London, Uxbridge UB8 3PH, UK; aeman.zahra@brunel.ac.uk (A.Z.); Rachel.Kerslake3@brunel.ac.uk (R.K.); 2 Warwickshire Institute for the Study of Diabetes, Endocrinology and Metabolism (WISDEM), University Hospitals Coventry and Warwickshire NHS Trust, Coventry CV2 2DX, UK; kyrouj@gmail.com (I.K.); harpal.randeva@uhcw.nhs.uk (H.S.R.); 3 Warwick Medical School, University of Warwick, Coventry CV4 7AL, UK; 4 Centre for Sport, Exercise and Life Sciences, Research Institute for Health & Wellbeing, Coventry University, Coventry CV1 5FB, UK; 5 Aston Medical Research Institute, Aston Medical School, College of Health and Life Sciences, Aston University, Birmingham B4 7ET, UK; 6 Laboratory of Dietetics and Quality of Life, Department of Food Science and Human Nutrition, School of Food and Nutritional Sciences, Agricultural University of Athens, 11855 Athens, Greece

**Keywords:** endocrine-disrupting chemicals, EDC, Bisphenol A, BPA, ovary, ovarian cancer

## Abstract

Endocrine-disrupting chemicals (EDCs), including the xenoestrogen Bisphenol A (BPA), can interfere with hormonal signalling. Despite increasing reports of adverse health effects associated with exposure to EDCs, there are limited data on the effect of BPA in normal human ovaries. In this paper, we present a detailed analysis of the transcriptomic landscape in normal Human Epithelial Ovarian Cells (HOSEpiC) treated with BPA (10 and 100 nM). Gene expression profiles were determined using high-throughput RNA sequencing, followed by functional analyses using bioinformatics tools. In total, 272 and 454 differentially expressed genes (DEGs) were identified in 10 and 100 nM BPA-treated HOSEpiCs, respectively, compared to untreated controls. Biological pathways included mRNA surveillance pathways, oocyte meiosis, cellular senescence, and transcriptional misregulation in cancer. BPA exposure has a considerable impact on 10 genes: *ANAPC2*, *AURKA*, *CDK1*, *CCNA2*, *CCNB1*, *PLK1*, *BUB1*, *KIF22*, *PDE3B*, and *CCNB3*, which are also associated with progesterone-mediated oocyte maturation pathways. Future studies should further explore the effects of BPA and its metabolites in the ovaries in health and disease, making use of validated in vitro and in vivo models to generate data that will address existing knowledge gaps in basic biology, hazard characterisation, and risk assessment associated with the use of xenoestrogens such as BPA.

## 1. Introduction

Endocrine-disrupting chemicals (EDCs) are widespread in the environment, from manufacturing to packaging and waste materials. Once in the environment, EDCs can accumulate throughout food chains and have the potential to disturb the normal endocrine functions of organisms [1,2]. Notably, EDCs are not readily metabolised by the body and accumulate within tissues due to their lipophilic properties, whilst this accumulation appears to be associated with a diverse spectrum of health issues [1,3]. 

Bisphenol A (BPA) is one of the most common and thoroughly studied EDCs, representing one of the highest manufactured chemicals globally [4,5]. The world production of BPA is estimated to reach over 7348 K tonnes annually by the end of 2023 [6]. BPA is widely used as a monomer to manufacture polycarbonate plastics and metal tins [7]. Accordingly, due to its presence in numerous commercial products—ranging from food packaging and food contact materials to thermal paper, cosmetics, dust and medical materials—humans are exposed to BPA on a daily basis [8]. The most common routes of human BPA exposure are inhalation, ingestion, and transdermal contact [9,10]. Of note, studies have shown that the levels of accumulated BPA within human adipose tissue lie between 8 nM and 80 nM [11]. Interestingly, infants aged 0–6 months that are exclusively fed with canned formula milk and using polycarbonate bottles have been estimated to have the highest BPA exposure [12,13]. Such exposure during the developmental stages makes humans particularly vulnerable to harmful effects of BPA and other EDCs since their effects occur during crucial stages of organogenesis and tissue development that are normally mediated/controlled by finely regulated molecular and biochemical processes [14]. 

At a molecular level, BPA mimics the hormone estrogen and can therefore interfere with estrogen signalling pathways [8,15,16]. The estrogen signalling pathway is controlled at the genomic level by estrogen receptors ERα and ERβ; the non-genomic level by G protein-coupled receptor 30, GPR30; or GPER [17]. Particularly, GPR30 plays a role in reproductive physiology [18] and in the stimulation of female reproductive neoplasms, specifically breast, endometrial, ovarian, and cervical [19]. Accordingly, several studies have raised the possibility of a direct link between BPA and hormone-dependent cancers (e.g., ovarian, breast, and prostate cancer) [20,21]. 

Over the past decade, there have been a number of studies pointing toward the adverse effects of BPA on female reproductive tissues in both human and animal studies. For example, BPA was found to exert effects on normal ovaries, with oocyte abnormalities noted in adult mice exposed to BPA [22], whereas rats exposed to BPA (10 mg·kg^−1^·day^−1^) accelerated pubertal development [23]. BPA also disrupts and increases oocyte degeneration in human oocytes and meiotic maturation [24]. In a recent study of 106 women undergoing in vitro fertilisation–embryo transfer (IVF-ET), a significant decrease in embryo implantation rate was observed in the group with elevated BPA levels [25]. In the same study, BPA induced autophagy in human granulosa cells, involving the mTOR pathway. In a zebrafish model, low-dose exposure to BPA caused changes in oxidative stress response and metabolic fluxes that can potentially induce the premature maturation of oocytes [26]. Alterations in other reproductive tissues were also noted upon treatment with BPA. For example, prenatal BPA exposure in rhesus macaque altered the percentage of different cells in the fetal oviduct [27], and exposure of albino rats to BPA led to the degeneration of the vaginal epithelium [28]. In addition, CD1 mice treated with BPA exhibited uterine polyps and sarcoma of the uterine cervix [29]. In a recent meta-analysis and systematic review, an association was shown between higher BPA exposures and a higher risk of preterm birth [30]. Moreover, our group showed that BPA can drive post-translational modifications, alter cell proliferation, and induce gene changes in a placental in vitro model [31]. In terms of large-scale human epidemiological data on the effects of BPA, they are limited (source: epa.gov, accessed on 27 March 2022). 

In this paper, we present an analysis of the genomic activity landscape in normal Human Epithelial Ovarian Cells (HOSEpiC) under the influence of BPA. We found that 76 genes are solely dysregulated (*p* < 0.05) in the presence of the environmental doses of BPA, and we proceeded to functionally annotate them and evaluate their potential as disease drivers.

## 2. Results

### 2.1. Identification of Differentially Expressed Genes (DEGs)

**Figure 1:**
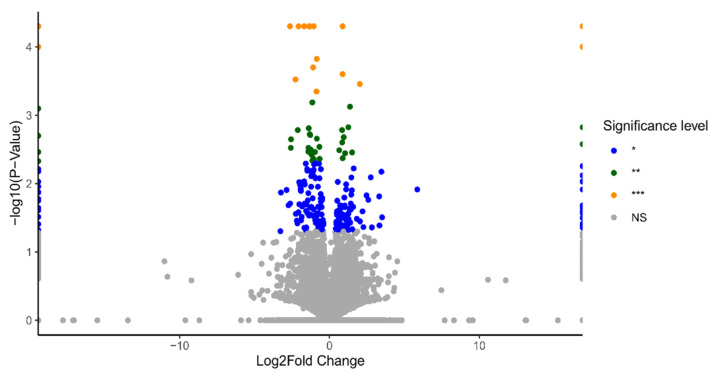
Volcano plot presenting all the differentially expressed genes (DEGs) upon the treatment of BPA 10 nM. Significance level for these gene was set as (blue dots * *p*-value < 0.05, green dots ** *p*-value < 0.005, orange dots *** *p*-value < 0.0005, and grey dots for no significant change (NS).

**Figure 2:**
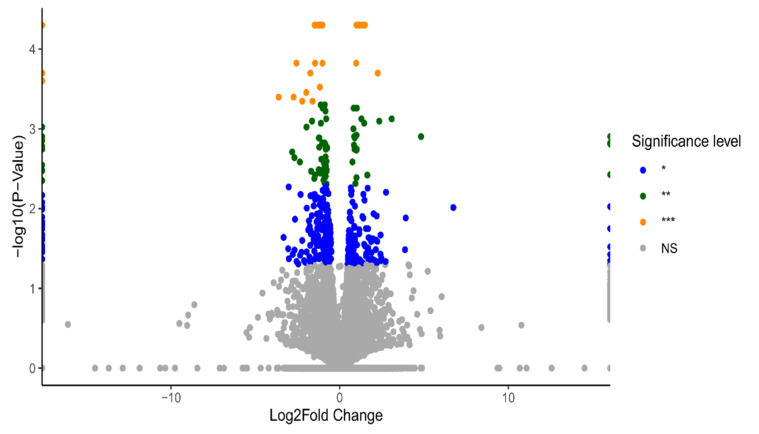
Volcano plot presenting the differentially expressed genes (DEGs) upon the treatment of BPA at 100 nM. Significance level for these gene was set as: blue dots * *p*-value < 0.05, green dots ** *p*-value < 0.005, orange dots *** *p*-value < 0.0005, and grey dots for no significant change (NS).

HOSEpiC cells were treated with 10 nM and 100 nM BPA treatments for 24 h (3 biological replicates), and DEGs were identified using the multiple-testing module from Cuffdiff, with significant changes defined based on a *p*-value < 0.05. To visualise the gene-expression profiles across all doses and replicates, volcano plots were generated using information from the statistical significance data (*p*-value) and the magnitude of change (fold change) between two conditions: BPA 10 nM vs. control (Figure 1) and BPA 100 nM vs. control (Figure 2).

**Figure 3:**
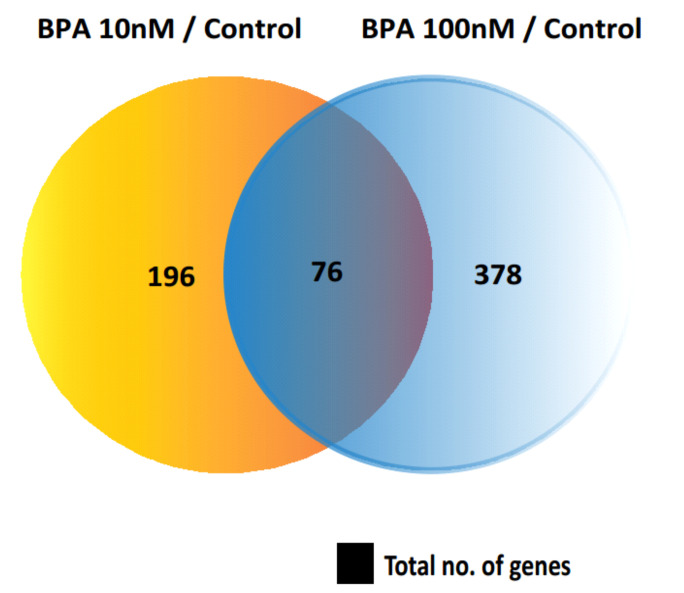
Venn diagram indicates the overlap of differentially expressed genes (DEGs) in cells treated with 10 nM and 100 nM BPA compared with the control group.

In total, 272 DEGs were identified in 10 nM BPA-treated HOSEpiC samples and 454 DEGs in the 100 nM BPA-treated ones compared to the control group. Among the DEGs identified in both groups, 76 genes were found to be commonly dysregulated irrespective of the level of BPA exposure (Figure 3). 

**Figure 4:**
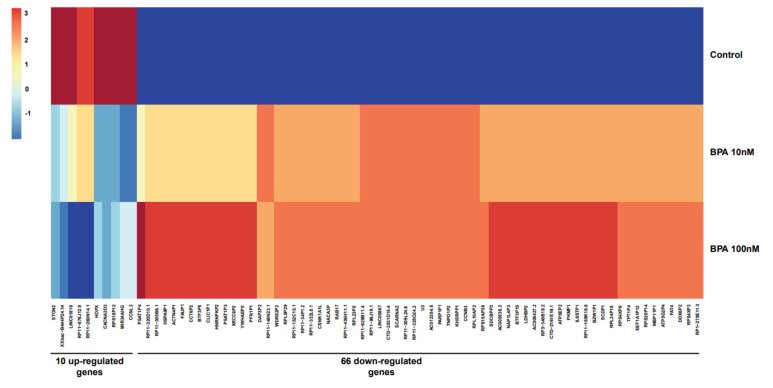
Heatmap reproduced expression profile for genes differently regulated (*p* < 0.05) over two used BPA doses (10 nM and 100 nM) and control group. Dark blue indicates low expression, and deep red indicates high expression.

Furthermore, hierarchical clustering in the 76 differential gene-expression profiles for 10 nM and 100 nM BPA treatment demonstrated similarities in both upregulated (*n* = 10) and downregulated (*n* = 66) DEGs compared to non-treated (control group) HOSEpiC cells (Figure 4). The heatmap depicts the expression of each gene in all the samples from the different groups in the experiment (BPA 10 nM, BPA 100 nM, and untreated (control) groups).

### 2.2. Functional Annotation Analysis of the DEGs

**Figure 5:**
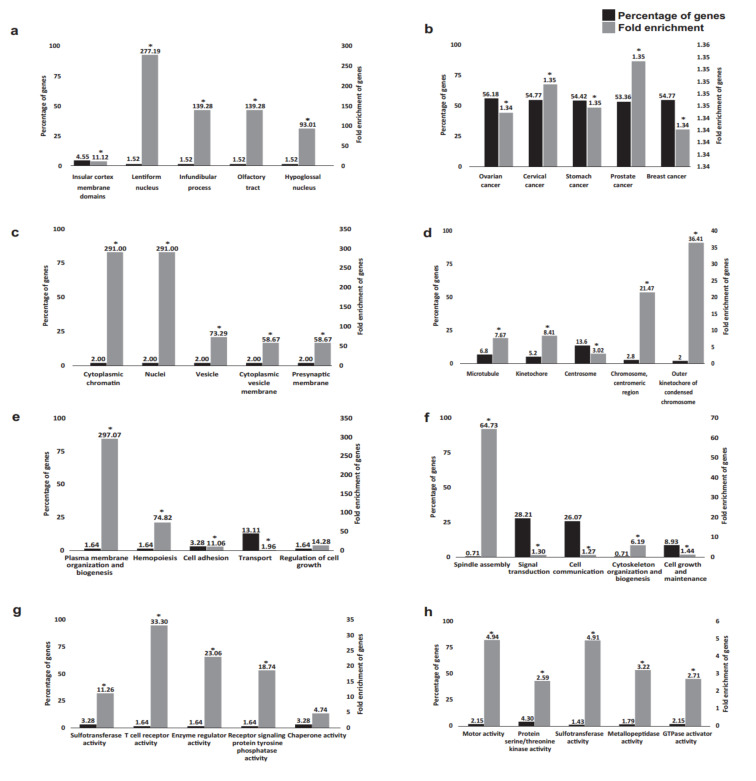
The functional enrichment in Gene Ontology terms in BPA 10 nM exposure DEGs (**a**,**c**,**e**,**g**) and BPA 100 nM exposure DEGs (**b**,**d**,**f**,**h**) in relation to site of expression (**a**,**b**), cellular components (**c**,**d**), biological processes (**e**,**f**), and molecular functions (**g**,**h**). * *p* < 0.05.

Next, DEGs with cut-off criteria of *p* < 0.05 and [Log2FC] > 1 were selected for subsequent functional analysis (Figure 5). In total, 70 out of 196 DEGs by BPA 10 nM exposure were previously described in the literature and were identified by the functional annotation FunRich database. An additional 286 out of 378 DEGs were recognised by the functional annotation FunRich database for the 100 nM BPA exposure.

Gene Ontology (GO) analysis indicated that the majority of genes affected by exposure to 100 nM BPA are also dysregulated in various female cancers (specifically, 159 genes in ovarian cancer and 155 genes in cervical and breast cancer). Notably, the current literature describes the impact of BPA exposure for only 2 genes out of the 76 identified by our study (Supplementary Figure S1).

**Figure 6:**
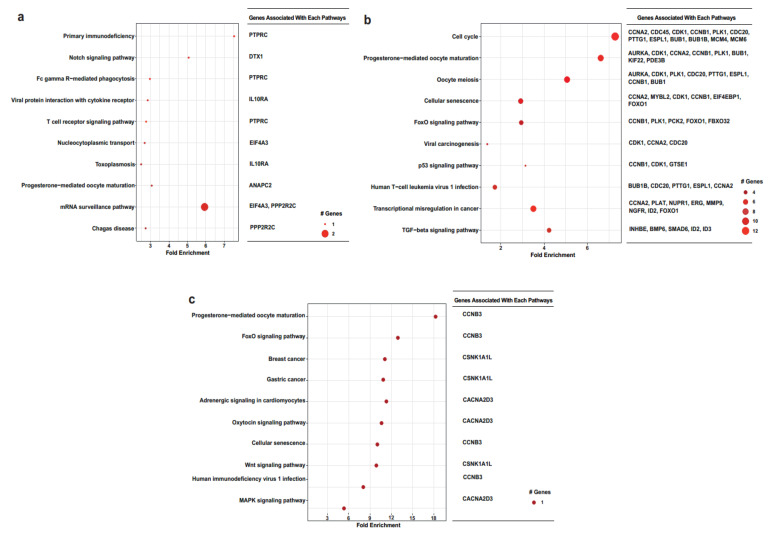
Biological pathways associated with the exposure of the different environmental doses of BPA (10 nM (**a**) and 100 nM (**b**)) dysregulated genes, along with shared common DEGs of these two doses (**c**).

Furthermore, we looked at identifying the biological pathways associated with the three sets of DEGs: 10 nM BPA (*n* = 78)-specific, 100 nM BPA (*n* = 289)-specific, and common DEGs over these two doses (*n* = 13) (Figure 6a–c). 

The results show that BPA exposure has a considerable impact on 10 genes: *ANAPC2*, *AURKA*, *CDK1*, *CCNA2*, *CCNB1*, *PLK1*, *BUB1*, *KIF22*, *PDE3B*, and *CCNB3*, which are also associated with progesterone-mediated oocyte maturation pathways. Studies have suggested that exposure to BPA may cause an increase in meiotic disturbances in mice, such as aneuploidy in oocytes [32,33]. It is well documented that exposure to BPA in the prenatal period is associated with cystic endometrial hyperplasia, ovarian cysts, aneuploidy in oocytes, and a reduction in the primordial pool of follicles in mouse ovaries, indicating an association between BPA and the increased proliferation of ovarian cells mediated by estrogenic pathway [33,34,35].

**Figure 7:**
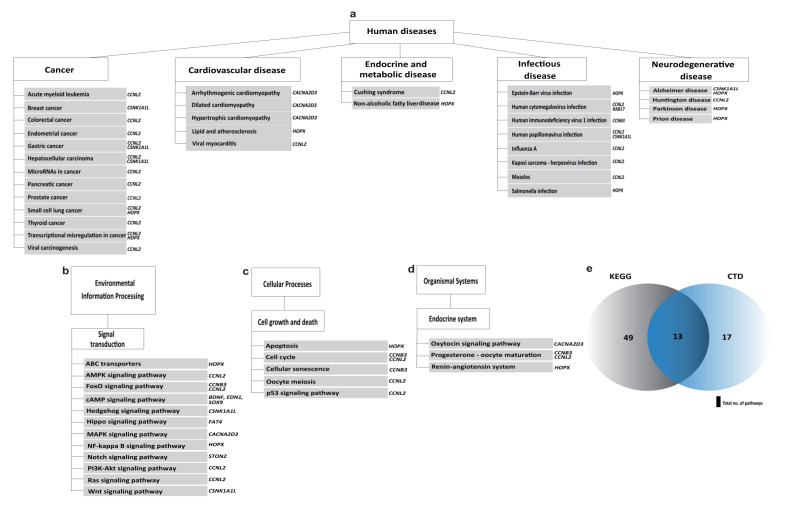
Biological pathways associated with BPA-dysregulated genes in humans. (**a**) Human-disease-associated pathways. (**b**) Environmental information processing pathways. (**c**) Cellular-processes-associated pathways. (**d**) Endocrine-system-associated pathways. (**e**) Venn diagram presenting the common pathways in KEGG- and BPA-impacted pathways reported in CTD. Genes that affect each pathway are shown on the right corner of each block.

Finally, we investigated biological pathways from the Kyoto Encyclopedia of Genes and Genome (KEGG) and Comparative Toxicogenomics Database (CTD) using the shared DEGs in the two used BPA doses (Figure 7). Accordingly, we found that the DEGs are mainly involved in pathways associated with human diseases, particularly cancer (Figure 7a) and various infectious diseases (viral, bacterial, and parasitic); environmental information processing (Figure 7b); cellular processes, including cell growth and death (Figure 7c); and organismal systems, i.e., the endocrine system (Figure 7d). Furthermore, 30 pathways have been previously described in the literature as being impacted by BPA exposure (Figure 7e). Out of those 30 pathways, 13 pathways (Table 1) were common between the 2 databases.

## 3. Discussion

In the present paper, we provide evidence of the impact that BPA can have across the ovarian transcriptome using a primary ovarian cell line (HOSEpiC) as an experimental model. In total, 272 DEGs were identified when cells were treated with 10 nM BPA, whereas at 100 nM, 454 DEGs were identified, out of which 76 were commonly regulated. 

In accordance with differences in DEGs, functional analysis of expression site, cellular components, biological processes, and molecular function revealed dose-specific effects. For example, a much higher percentage of genes was identified in cells treated with 100 nM BPA with enrichment primarily around gynaecological malignancies, including ovarian cancer, in terms of site of expression. Indeed, we and others have recently discussed the potential involvement of BPA in ovarian cancer aetiopathogenesis [21,33,36]. In terms of cellular components, both BPA concentrations used appear to modulate a wide repertoire, ranging from cytoplasmic chromatin and nuclei at 10 nM and chromosomal regions at 100 nM. Previous studies in mouse spermatozoa revealed that exposure to BPA led to incomplete chromatin condensation, as well as abnormalities in acrosome formation [37]. Similarly, in male zebrafish, when exposed to BPA (100 µg/L), sperm chromatin fragmentation was increased; hence, the authors suggested that “BPA male exposure jeopardises embryonic survival and development” [38]. Moreover, when rat ovaries were treated with BPA in vitro, this led to a reduction in primary and secondary follicle numbers with evident DNA damage (ovotoxicity) [39]. In line with such data, our data are also suggestive of BPA exerting similar deleterious effects in human ovaries, affecting chromatin reorganisation. 

Furthermore, there were also non-overlapping modalities in biological processes. For example, previous studies have shown that the plasma membrane organisation and biogenesis were enriched at 10 nM BPA, whereas spindle assembly demonstrated the highest fold enrichment at 100 nM of BPA treatment. Notably, the speed assembly checkpoint is vital for the safeguarding of the transmission of sister chromatids to two daughter cells, monitoring chromosomal segregation [40]. In addition, Kim et al. showed that BPA interferes with spindle microtubule attachment to kinetochores during the process of mitosis, ultimately driving tumorigenesis by enhancing chromosome instability in vitro [41]. Of note, there is a correlation between spindle assembly checkpoint protein expression and a shorter time of ovarian cancer recurrence [42]. Molecular functions depicted a similar diversity, with T-cell-receptor activity being the most enriched function at 10 nM BPA and motor and sulfotransferase activity at 100 nM of treatment. Dysregulation of T-cell receptors can give rise to a number of diseases, given that adaptive immunity will be compromised [43]. Previous studies have also shown that prenatal exposure to BPA in mice resulted in altered immune response involving T-helper 1 (Th1) cells [44]. On the other hand, a number of sulfotransferases (SULTs) are highly expressed in the human ovary [45] and can be a potential therapeutic target for ovarian cancer.

We then took a “deep dive” into the biological pathways for all three sets of DEGs, where we showed that the most enriched pathway at 10 nM of BPA treatment was that of mRNA surveillance, a pathway crucial for the quality of mRNA by degrading harmful RNAs [46]. Mutations or dysregulation of this pathway can give rise to various diseases. Here, we found that the genes involved include EIF4A3 and PPP2R2C. To the best of our knowledge, this is the first time that it has been shown that these two genes are dysregulated by BPA at the normal ovarian level. In ovarian cancer, there is upregulation of EIF4A3 [47], whereas suppression of PPP2R2C leads to ovarian cancer cell proliferation [48]. In cells treated with 100 nM of BPA, the cell cycle was the most enriched modality, with some of the identified genes playing a crucial role in the ovaries. For example, when CDK1 activity is inhibited by phosphorylation, it leads to the prolonged arrest of prophase-I in female germ cells, thus underpinning its importance for the female reproductive lifespan [49]. BUB1 (a mitotic checkpoint serine/threonine kinase) is another identified gene within our data that is involved in the cell cycle. Of note, Leland et al. showed that there is a link between inherited aneuploidy in female germ cells and dysfunction of BUB1, which can ultimately lead to loss of pregnancy [50]. 

Interestingly, a common pathway that was enriched by both concentrations of BPA was that of progesterone-mediated oocyte maturation. Oocyte maturation, along with embryo development, is controlled by steroid hormones, including progesterone [51]. CCNA2 and CCNB3 are two DEGs affected by BPA. CCNA2, in particular, is of importance since when conditional knockout mice for CCNA2 were generated, the female mice were infertile [52,53]. Similarly, CCNB3-deficient female mice are also sterile [54]. In another study, a CCNB3 mutation affected the metaphase–anaphase transition in oocyte meiosis I, again leading to infertility [55]. 

We acknowledge certain limitations of our study, including utilising a singular primary ovarian cell line as a relevant in vitro model and choosing to assess only two concentrations of BPA. However, the utilised doses reflect the range of BPA environmental doses. Future studies should concentrate on expanding the use of both in vitro and ex vivo models (including 3D cultures and ovarian explants), as well as discerning whether BPA effects are mediated via canonical nuclear estrogen receptors or membrane-bound GPR30. Finally, our RNA sequencing data can be further validated by using RT-qPCR in addition to Western blot analysis to measure gene and protein level changes exerted by the identified DEGs. 

Ten years ago, in a foetal rhesus monkey model, BPA exposure was shown to alter oogenesis and follicle formation [56]. Since then, a number of studies have argued that the human ovary can also be a target for endocrine disruption [57]. Our study provides a novel insight into the transcriptome changes at the ovarian level upon exposure to BPA. We hope these data will be used as a starting point for future in vitro and in vivo studies assessing the impacts of EDCs on health and disease. It should be noted that the primary route of human exposure to BPA for most is through the diet, as this EDC leaches from drink and food containers, particularly when they are heated. Alternative—but minor—routes of exposure include dental sealants, inhalation, dermal absorption, and maternal exposure [58,59,60,61]. These diverse routes of exposure present certain challenges in how to assess effects in vitro, ex vivo, and in vivo. For example, 3D ovarian cultures might be a more physiologically relevant system than 2D, where the effect of BPA can be studied on spheroids of primary ovarian cells as well as in different ovarian cancer cells in an attempt to understand the implications of EDCs in the tumour microenvironment [62]. Alternatively, ovarian tissue explants can be used as preclinical models [63]. This approach might give a better representation of the multicellular environment, and a number of readouts can be performed, including spatial transcriptomics and X-ray microtomography, which will provide even more information on the role of BPA. Alternatively, in vivo models of exposure can also be used, but for those to take place, research groups must adhere to the principles of the 3Rs (Replacement, Reduction, and Refinement). Over the past decade (2012–2022), 2101 manuscripts have been published on “BPA treatment” in animal models (source: PubMed). However, the key question is how relevant are these models to ovarian physiology in the context of EDC exposure? Therefore, a number of considerations must be made in order to identify the right model that will mimic EDC exposure in humans [64]. Finally, when designing such experiments, the effects of multiple xenoestrogens should be taken into consideration since they can have a tremendous additive impact, altering hormonal actions [65].

To summarise, with the current study, we have added to the existing literature by providing a novel insight into the effects of BPA in the human ovary, which can potentially compromise specific signalling pathways, leading to alterations in reproductive physiology. Future studies using 3D cell cultures/spheroids and ex vivo and in vivo models will further address gaps in knowledge of the effect of BPA (and other EDCs or their mixtures) at the ovarian level. Collectively, emerging studies will play a pivotal role in the legislation around EDCs. For example, the European Food Safety Authority (EFSA) re-evaluated the risks associated with BPA and proposed to considerably lower the tolerable daily intake (TDI) compared to its previous assessment in 2015, from 4 µg/kg bw/day to 0.04 4 µg/kg bw/day (source: efsa.europa.eu, accessed on 27 March 2022). Therefore, particular emphasis should be given to future studies that will elucidate the precise signalling mechanisms involved in endocrine disruption in reproductive organs. Moreover, consideration should also be given to the role of analogues to BPA (e.g., BPS) and their mixtures in health and disease. 

## 4. Materials and Methods

### 4.1. Cell Culture

Primary normal ovarian epithelial cells, HOSEpiC (#7310), acquired at passage 1 from ScienCell Ltd., were cultured with Ovarian Epithelial Cell Medium (OEpICM), supplemented with 1% Ovarian Cell Growth Supplement (ScienCell Ltd., Carlsbad, CA, USA), 1% penicillin–streptomycin, and 10% FBS (Thermo Fisher Scientific, Loughborough, UK) in Poly-L-Lysine (ScienCell Ltd., Carlsbad, CA, USA)-coated T25 flasks. Prior to cell seeding, all flasks and plates were treated with 5 ug/mL Poly-L-Lysine in sterile de-ionised water for 1 h at 37 °C, washed with de-ionised water, and returned to the incubator for an additional hour to dry. Cell count and viability were carried out manually using a Neubauer chamber and Trypan blue (Invitrogen; Thermo Fisher Scientific, Loughborough, UK) exclusion method. Adherent cells were detached using TrypLE express (Thermo Fisher Scientific, Loughborough, UK). At passage 2, cells were transferred to a T75 flask before seeding in 6-well plates at a density of 0.3 × 10^6^. At a confluence of 80%, media was replenished, and cells were treated with 10 nM and 100 nM of BPA (Sigma-Aldrich, St. Louis, MO, USA) in triplicate (detail is given below). 

### 4.2. RNA Extraction

Samples were extracted, and the experiments were arrested at 24 h. Media were removed, and cells were washed with 500 μL of cold sterile PBS (Thermo Fisher Scientific, Loughborough, UK). RNA isolation was achieved using Qiagen RNeasy extraction kit (Qiagen, Manchester, UK); following the manufacturer’s instruction, 40 μL of RNA was eluted. Samples were then stored at −80 °C prior to shipment for sequencing. 

### 4.3. RNA-Sequencing (RNA-Seq), Data Generation

The samples were sequenced using Illumina sequencing, which resulted in taking the average of reads for each experimental replicate of the three experiments (Table 2). 

RNA-seq processing pipeline was designed using TopHat2 (v.2.1.1) tool to align RNA-Seq reads to the human reference genome GRCH38 (hg19) using the ultra high-throughput short read aligner Bowtie2 (v.2.2.6). Next, Samtools (v.0.1.19) was used to merge all experimental replicates and to view and select high-quality mapped reads (minimum quality threshold was set at 30). Transcript assembly and expression quantification in each sample was conducted using Cufflinks (v.2.2.1). Finally, a differential expression profile between two experiments was obtained using Cuffdiff. 

### 4.4. Statistical RNA-Sequencing Analysis

All RNA-seq data processing, modelling, cleaning, visualising, and statistical analysis were conducted using R (v. 4.1.0, The R Foundation for Statistical Computing, Vienna, Austria) under R Studio desktop application (version 1.4.1717, RStudio, Boston, MA, USA). The Pearson correlation coefficient was calculated to estimate the correlation between genes based on their expression pattern in all the experiments. Student’s *t*-test was used to assess the statistical significance of the change of expression between two given states (e.g., BPA 10 nM vs. BPA 100 nM) with a significance threshold set at a *p*-value lower than 0.05. Volcano plots, heatmap, and Venn diagram were also generated using R. R package pathfindR was used for comprehensive identification of enriched pathways in omics data.

### 4.5. Functional Annotation

The shared differentially expressed genes (DEGs) from HOSEpiC samples treated with 10 nM BPA and 100 nM BPA in comparison with the control ethanol-treated samples were used for further functional annotation, as outlined below.

#### 4.5.1. KEGG Pathway Database

Pathway analysis of the DEGs was performed by quarrying the KEGG database (https://www.kegg.jp/kegg/pathway.html (accessed on 8 February 2022)). KEGG is a collection of manually drawn pathway maps representing the current knowledge base of the molecular interaction, reaction, and regulation networks for human diseases, environmental information processing, organismal systems, and drug development.

#### 4.5.2. Comparative Toxicogenomics Database (CTD)

In order to understand how environmental exposures affect human health, the CTD (http://ctdbase.org/; accessed on 8 February 2022) was used since it provides manually curated information about small molecule chemicals–gene and small molecule chemicals–disease interactions, and gene–disease pathway relationships.

#### 4.5.3. Functional Analysis

The genes were functionally characterised using the Gene Ontology (GO) database [66], as recorded in FunRich (version 3.1.3) software [67]. The enrichment of the GO terms related to biological processes, biological pathways, molecular functions, and expression sites was computed. A threshold *p*-value of 0.05 was used to ascertain the statistical significance of the results.

#### 4.5.4. The Gene Ontology Consortium

GO Consortium resource (http://geneontology.org/ accessed on 5 March 2022) was used to develop a comprehensive, computational model of biological systems, ranging from the molecular to the organism level. The statistical significance of the results was obtained by threshold *p*-value of 0.05. Currently, the GO includes experimental findings from over 150,000 published papers, represented as over 700,000 experimentally supported annotations.

## 5. Conclusions 

In the present paper, we provide evidence of the impact that BPA can have across the ovarian transcriptome using a primary ovarian cell line (HOSEpiC) as an experimental model. Future studies should further explore the changes that BPA and other common EDCs can elicit within the ovaries at gene, protein, and metabolic levels, subsequently addressing existing knowledge gaps in basic biology, hazard characterisation, and risk assessment associated with the use of xenoestrogens such as BPA at the ovarian level.

## 6. Patents

No patents resulted from the work reported in this manuscript.

## Figures and Tables

**Table 1:** In existing literature, 13 common pathways have been previously described as being impacted by BPA exposure with associated DEGs from this study.

Pathways	Associated Genes
Arrhythmogenic right ventricular cardiomyopathy	CACNA2D3
Breast cancer	CSNK1A1L
Cell cycle	CCNB3, CCNL2
Dilated cardiomyopathy	CACNA2D3
FoxO signalling pathway	CCNB3, CCNL2
Hedgehog signalling pathway	CSNK1A1L
Hippo signalling pathway	FAT4
Hypertrophic cardiomyopathy (HCM)	CACNA2D3
MAPK signalling pathway	CACNA2D3
Oxytocin signalling pathway	CACNA2D3
Progesterone-mediated oocyte maturation	CCNB3, CCNL2
p53 signalling pathway	CCNB3, CCNL2
Wnt signalling pathway	CSNK1A1L

**Table 2:** Total number of reads. For paired-end sequencing, these values refer to the sum of read 1 and read 2.

Samples	Total Reads
Control	75,835,336
BPA 10 nM	82,440,001
BPA 100 nM	65,361,410

## Data Availability

All data is publicly available from online repositories as indicated in the materials and methods section.

## Supplementary Materials

The following supporting information can be downloaded at: https://www.mdpi.com/article/10.3390/ijms23105334/s1.
