# Supplementary material for: Impact of Environmentally Relevant Concentrations of Bisphenol A (BPA) on the Gene Expression Profile in an In Vitro Model of the Normal Human Ovary
Source: Int J Mol Sci. 2022 May 10;23(10):5334. doi: 10.3390/ijms23105334 (PMC9141570; doi:10.3390/ijms23105334)
Supplement: Supplementary file 1 [file ijms-23-05334-s001.zip › ijms-1678405-supplementary.pdf]

Supplementary Materials

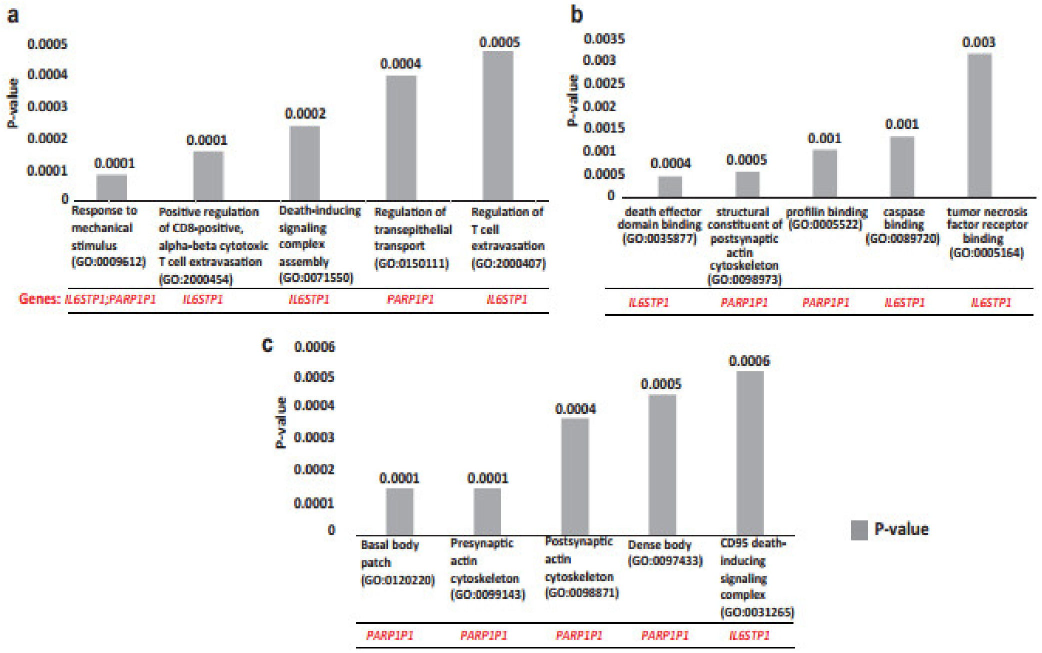

Figure S1. The functional enrichment in Gene Ontology terms in shared differentially expressed genes (DEGs) over the two used doses of BPA (10 nM and 100 nM) in relation to Biological processes (**a**), Molecular functions (**b**), and Cellular components (**c**).
